# (Wh)olistic (E)ndocannabinoidome-Microbiome-Axis Modulation through (N)utrition (WHEN) to Curb Obesity and Related Disorders

**DOI:** 10.1186/s12944-021-01609-3

**Published:** 2022-01-14

**Authors:** Jyoti Sihag, Vincenzo Di Marzo

**Affiliations:** 1 Faculty of Medicine, University of Laval, Quebec, Canada; 2 Faculty of Agriculture and Food Sciences, University of Laval, Quebec, Canada; 3 Canada Excellence Research Chair on the Microbiome-Endocannabinoidome Axis in Metabolic Health (CERC-MEND), University of Laval, Quebec, Canada; 4 University Institute of Cardiology and Pneumology, Quebec, Canada; 5 Institute of Nutrition and Functional Foods (INAF) and Centre Nutrition, Santé et Société (NUTRISS), University of Laval, Quebec, Canada; 6 Department of Foods and Nutrition, Chaudhary Charan Singh Haryana Agricultural University, Hisar, India; 7 Institute of Biomolecular Chemistry of the National Research Council (ICB-CNR), Naples, Italy; 8 Endocannabinoid Research Group, Naples, Italy; 9 Joint International Research Unit between the Italian National Research Council (CNR) and University of Laval, for Chemical and Biomolecular Research on the Microbiome and its impact on Metabolic Health and Nutrition (UMI-MicroMeNu), Quebec, Canada

**Keywords:** Endocannabinoids, Endocannabinoidome, Microbiome, Nutrition, Obesity

## Abstract

The discovery of the endocannabinoidome (eCBome) is evolving gradually with yet to be elucidated functional lipid mediators and receptors. The diet modulates these bioactive lipids and the gut microbiome, both working in an entwined alliance. Mounting evidence suggests that, in different ways and with a certain specialisation, lipid signalling mediators such as *N*-acylethanolamines (NAEs), 2-monoacylglycerols (2-MAGs), and *N*-acyl-amino acids (NAAs), along with endocannabinoids (eCBs), can modulate physiological mechanisms underpinning appetite, food intake, macronutrient metabolism, pain sensation, blood pressure, mood, cognition, and immunity. This knowledge has been primarily utilised in pharmacology and medicine to develop many drugs targeting the fine and specific molecular pathways orchestrating eCB and eCBome activity. Conversely, the contribution of dietary NAEs, 2-MAGs and eCBs to the biological functions of these molecules has been little studied. In this review, we discuss the importance of (Wh) olistic (E)ndocannabinoidome-Microbiome-Axis Modulation through (N) utrition (WHEN), in the management of obesity and related disorders.

## Introduction

The endocannabinoid (eCB) system is a crucial regulatory system involved in physiological homeostasis [1]. Humans as well as animals synthesize the endocannabinoids (eCBs) [2]. For the past two decades, the eCB system has extensively gained attention from scientists/researchers of varied scientific backgrounds, including physiology and neurology [3]. The term endocannabinoid, coined by Di Marzo and Fontana in the 1990s [4, 5], denotes endogenous metabolites capable of activating cannabinoid receptors. In fact, the eCB system was discovered secondary to the discovery of the psychotropic *Cannabis* component, ∆^9^-tetrahydrocannabinol (THC), which stimulates two G-protein coupled receptors (GPCRs), the cannabinoid receptors – CB1 and CB2, whose primary function is to be activated by endogenous compounds, the eCBs, derived from C20:4n6, i.e. arachidonoylethanolamide (AEA), commonly known as anandamide, and 2-arachidonoylglycerol (2-AG) [6]. CB1 receptors play pleiotropic homeostatic functions, such as neuromodulation in the brain, often resulting in appetite and energy intake stimulation [7, 8]. In addition, lately, the pharmaceutical importance of targeting the CB1 receptors has also been demonstrated in skeletal muscle cells [9–11]. Some of the observed effects were suggested to be regulated via downregulation of the pro-inflammatory cascades, particularly ablating the cytokine activity of interleukin-6 (IL-6) [9] and orphan nuclear receptor NR4A family – NR4A1, NR4A2, and NR4A3 [10]. On the contrary, CB2 receptors preferentially hold immunomodulatory functions, often resulting in the inhibition of inflammation [12].

The core eCB system consists of the two major eCBs, AEA and 2-AG, five enzymes for AEA and 2-AG biosynthesis and inactivation: i) *N*-acyl phosphatidylethanolamine phospholipase D (NAPE-PLD), ii) fatty acid amide hydrolase (FAAH), iii) diacylglycerol lipases α and β (DAGL α and β), and iv) monoacylglycerol lipase (MAGL); and the two cannabinoid receptors [13, 14]. However, AEA and 2-AG are accompanied in tissues by several congeners, i.e., the *N*-acylethanolamines (NAEs) and 2-monoacylglycerols (2-MAGs), respectively, as well as by other long-chain fatty acid amides, such as the *N*-acylamino acids (NAAs) [15]. These and other families of eCB-like lipids, together with their receptors, catabolic (often shared with the eCBs) and anabolic enzymes, and along with the eCB system, constitute the endocannabinoidome (eCBome) [1, 15].

**Fig. 1:**
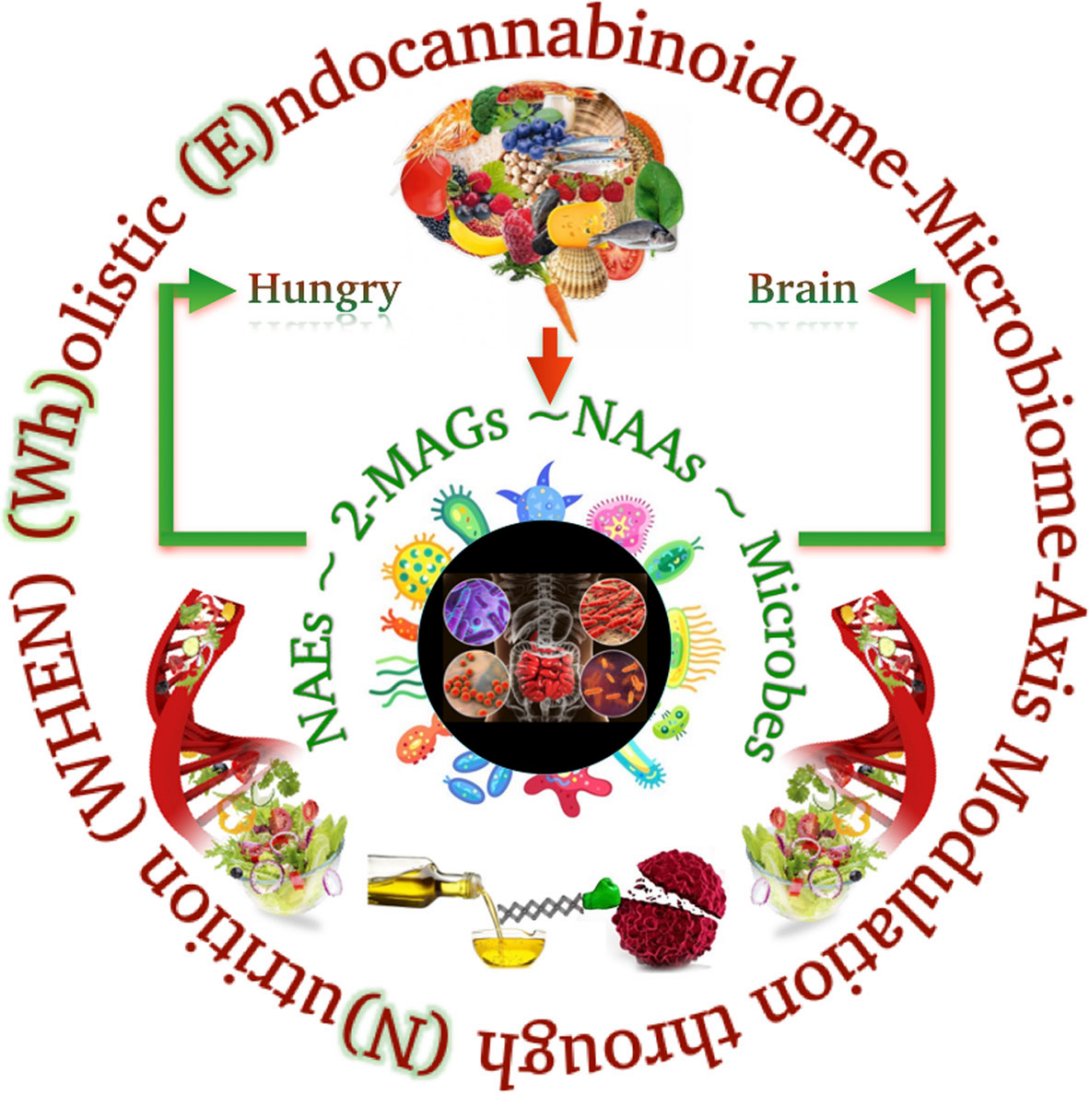
The (Wh) olistic (E)ndocannabinoidome-Microbiome-Axis Modulation through (N) utrition (WHEN) Model. How the hungry brain signals the homeostatic drive via the endocannabinoidome-gut microbiome axis. The consumption of a balanced diet leads to the endogenous synthesis of *N*-acyl-ethanolamines, 2-monoacylglycerols, *N*-acyl-amino acids, and hence also helps defining a healthy gut microbial ecosystem. The entwined matrix between the brain and gut, integrated with genetics, bioactive lipids, and lipid mediators 'interplay' acts on, among others, white and brown adipocytes, regulating energy homeostasis. **Note (alphabetical order):** 2-MAGs, 2-monoacylglycerols; NAAs, *N*-acyl-amino acids; NAEs, *N*-acyl-ethanolamines.

**Fig. 2:**
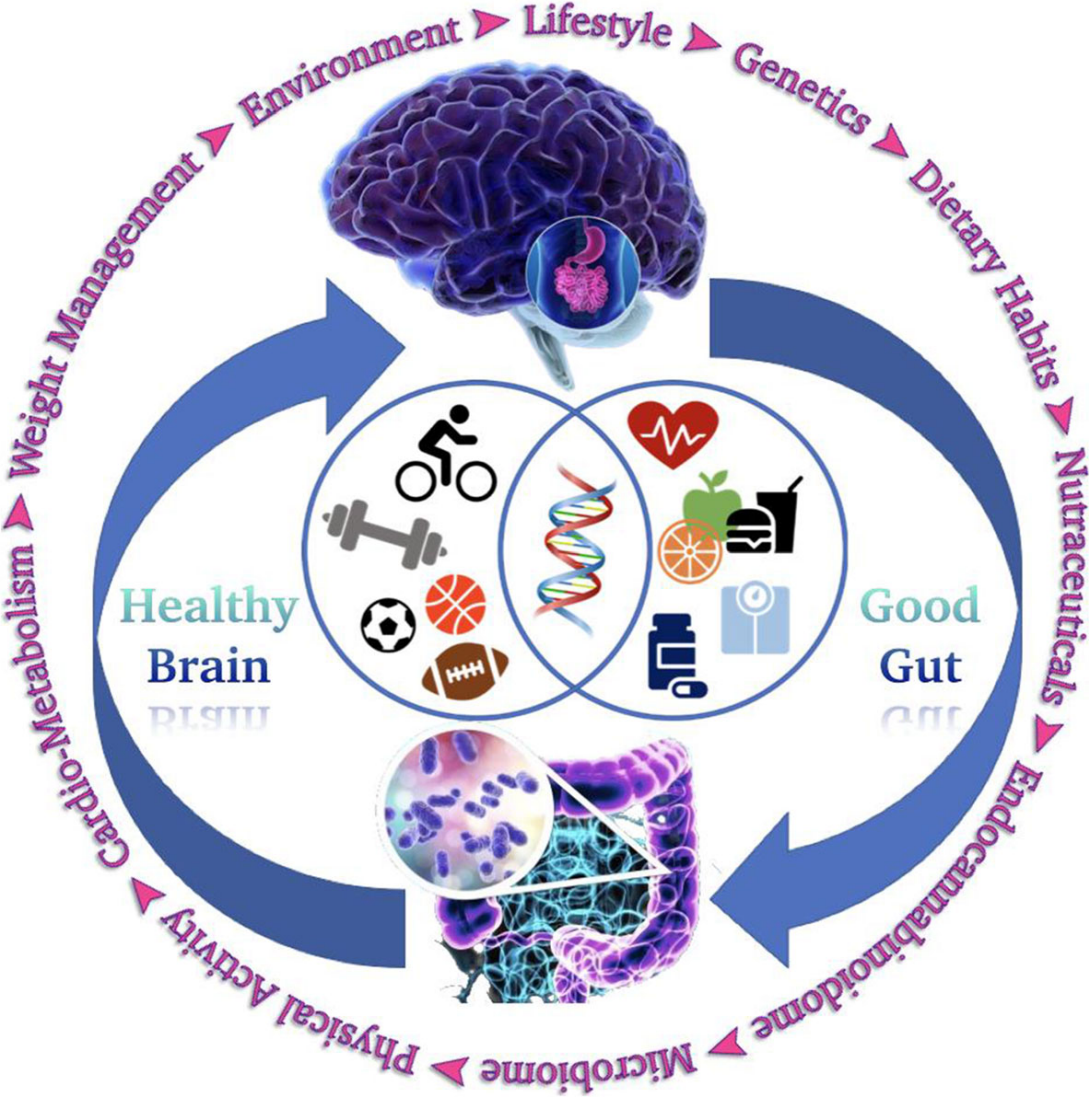
The human body and the 'two brains.' The central nervous system (brain 1) and enteric nervous system ('brain' 2). The unique neural integration between the two, post-consumption/ingestion of balanced diet, along with genetics, environmental and lifestyle factors, help attain a good gut and healthy brain, commanding overall wellness.

Excessive eCB signalling at CB1 receptors is emerging as one of the predisposing factors to hyperphagia, obesity and related disorders, such as type 2 diabetes (T2D), hepato-steatosis, peripheral organ inflammation, fibrosis, as well as to substance abuse and addiction [16]. Thus, the hyperactive eCB system has led to the targeting through antagonism or inverse agonism of CB1 receptors for the development not only of anti-obesity/T2D [17], but also of anti-nicotine and -alcohol addiction drugs [18]. Additionally, CB1 blockers have been beneficial for treating obesity-associated liver and kidney inflammation/fibrosis [19]. Despite showing positive results, these drugs were withdrawn from the market early on [20] because they also interfere with fundamental CB1 functions in the brain, such as, for instance, coping with stress, which, if impaired, may explain neuropsychiatric adverse effects. Even the development of suicidal tendencies is occasionally observed with CB1 antagonists/inverse agonists [21]. Instead, eCB signalling at CB2 receptors, on the one hand, and the activation of other eCBome receptors, such as peroxisome proliferator-activated receptors (PPAR)-α and -γ [22], orphan GPCRs including GPR18 [23], GPR55 [24], and GPR119 [25] and transient receptor vanilloid-type-1 (TRPV1) [26] channels by other NAEs [27], 2-MAGs [28] and NAAs [15], on the other hand, may counteract the obesity/T2D-worsening effects of excessive CB1 activation in peripheral organs, as well as its pro-addictive actions, without interfering with CB1 crucial functions in the brain. A very important feature of these eCB-like mediators is that for most of them, besides being available in small amounts in dietary fats and oils present in the whole foods [29], their endogenous production is also strongly influenced by the dietary intake of the corresponding fatty acids [30–33]. Additionally, the ingestion of plant-derived fats and oils with specific fatty acid compositions leads to not only the endogenous prevalence of some exclusive class of NAEs, 2-MAGs and NAAs, carrying anorexic or lipolytic properties, but also trigger the stimulation of healthy gut bacteria species such as *Bifidobacteria or Akkermansiaceae,* over others that may worsen, or even mediate, the dysmetabolic effects of CB1 receptors [34]. The present review aims to elaborate on prospective (Wh) olistic (E)ndocannabinoidome-Microbiome-Axis Modulation through (N) utrition (WHEN) strategies to counteract hyperphagia, obesity and related disorders by helping regulate energy intake and processing to maintain healthy body weight. We present the WHEN graphical model in Fig. 1, further expanded by a graphic illustration in Fig. 2.

## An array of bioactive lipids

The bioactive lipids of the eCBome are a class of endogenous fatty acid biomolecules characterised by a fatty acyl group linked to a primary amine metabolite with an amide bond or to glycerol with an ester bond [35]. These molecules are a major component of the eCBome and can be further sub-categorised into three major families: i) ethanolamine conjugates, ii) glycerol conjugates, and iii) amino acid conjugates [36]. This large "ome" of bioactive lipids comprises, among others, saturated, monounsaturated, and polyunsaturated NAEs, 2-MAGs and NAAs. The chief fatty acids utilized in the endogenous synthesis of these biomolecules are C14:0, C16:0, C18:0, C18:1, C18:2n6, C20:4n6, C18:3n3, C18:4n3, C20:4n6, C20:5n3, C22:5n3, and C22:6n3.

### *N*-acylethanolamines (NAEs)

*N*-acyl phosphatidylethanolamines (NAPEs), a minor family of membrane phospholipids, act as biosynthetic precursors of NAEs through the action, among others, of the NAPE-specific phospholipase D-like enzyme (NAPE-PLD) in animals and phospholipase β (PLD-β) in plants [37]. In animals, NAEs can be hydrolyzed to free fatty acids and ethanolamine by FAAH or *N*-acylethanolamine-hydrolyzing acid amidase (NAAA) [29] whereas, in plant tissues, only FAAH is present [38]. An isoform of FAAH, FAAH-2, is also expressed [39–41] in human tissues. Endogenous bioactive NAEs, apart from the eCB AEA, also include myristoylethanolamide (MEA), palmitoylethanolamide (PEA), stearoylethanolamide (SEA), oleoylethanolamide (OEA), linoleoylethanolamide (LEA), ⍺-linolenoylethanolamide (ALEA), steariodonoylethanolamide (STEA), eicosapentaenoylethanolamide (EPEA) docosapentaenoylethanolamide (DPEA) and docosahexaenoylethanolamide (DHEA), whose proposed receptors, when known, are summarized in Table 1.

**Table 1:** Lipid mediators and their subsequent action on enzymes and receptors involved in the endocannabinoid system and endocannabinoidome.

Protein	Names*	Endocannabinoid system		Endocannabinoidome		
		Lipid signalling mediators				
		NAEs	2-MAGs	NAEs	2-MAGs	NAAs
Enzymes						
Anabolic	NAPE-PLD	AEA	–	PEA, OEA, LEA and others	–	–
	ABHD4	AEA	–	PEA, OEA, LEA and others	–	–
	GDE1	AEA	–	PEA, OEA, LEA and others	–	–
	DAGL-⍺/β	–	2-AG	PEA, OEA, LEA and others	2-OG, 2-LG and others	–
Catabolic	FAAH	AEA	2-AG	PEA, OEA, LEA, DHEA	–	NOleG, NAraG
	MAGL	–	2-AG	–	2-OG, 2-LG and others	–
	ABHD6	–	2-AG	–	2-OG, 2-LG and others	–
	COX-2	AEA	2-AG	–	–	C20:4n6 amides
	ABHD12	–	2-AG	–	2-OG, 2-LG and others	–
Cannabinoid receptors	CB1	AEA	2-AG	–	–	–
	CB2	AEA	2-AG	EPEA, DHEA	–	–
G protein-coupled receptors	GPR6	–	–	–	–	NAraG
	GPR18	–	–	–	–	NAraG
	GPR55	AEA	2-AG	PEA	–	–
	GPR110	–	–	DHEA	–	–
	GPR119	–	–	OEA, LEA	2-OG, 2-LG	–
Peroxisome proliferator-activated receptors	PPAR-⍺	–	–	PEA, OEA	2-PG	NOleA, NOleG
	PPAR-γ	AEA, DHEA	–	–	–	–
Transient receptor potential channels	TRPV1	AEA	2-AG	OEA, LEA, DHEA	2-OG, 2-LG	NATaus
	TRPV2	–	–	OEA, LEA, as antagonists	–	–
	TRPV3	–	–	–	–	–
	TRPV4	–	–	–	–	NATaus
	TRPA1	–	–	–	–	–
	TRPM8	AEA, as antagonist	2-AG, as antagonist	–	–	–

**Note (alphabetical order):** *Protein names are ordered in the rank of importance. 2-AG, 2-arachidonoylglycerol; 2-LG, 2-linoleoylglycerol; 2-MAGs, 2-monoacylglycerols; 2-OG, 2-oleoylglycerol; 2-PG, 2-palmitoylglycerol; ABHD4, 6, 12, ⍺/β-hydrolases 4, 6, 12; AEA, arachidonoylethanolamide; CB1, 2, cannabinoid receptors1, 2; COX-2, cyclooxygenase-2; DAGL-⍺/β, diacylglycerol lipase-⍺/β; DHEA, docosahexaenoylethanolamide; EPEA, eicosapentaenoylethanolamide; FAAH, fatty acid amide hydrolase; GDE1, glycerophosphodiester phosphodiesterase 1; GPR6, 18, 55, 110, 119, G protein-coupled receptors6, 18, 55, 110, 119; LEA, linoleoylethanolamide; MAGL, monoacylglycerol lipase; NAAs, *N*-acyl-amino acids; NAEs, *N*-acyl-ethanolamines; NAPE-PLD*, N*-acyl phosphatidylethanolamine phospholipase D; NAraG, *N-*arachidonoyl-glycine; NATaus, *N*-acyl taurines; NOleA, *N*-oleoyl-alanine; NOleG, *N-*oleyl-glycine; OEA, oleoylethanolamide; PEA, palmitoylethanolamide; PPAR-⍺/γ, peroxisome proliferator-activated receptors-alpha/gamma; TRPA1, transient receptor potential ankyrin 1; TRPM8, transient receptor potential melastatin 8; TRPV1, 2, 3, 4; transient receptor potential vanilloid 1, 2, 3, 4.

### 2-monoacylglycerols (2-MAGs)

2-MAGs are glycerol derivatives of fatty acids through ester bond formation [42]. They share the same metabolic pathways as 2-AG; however, they act on a distinct set of receptors [30, 36]. All receptors, where known, are summarized in Table 1. 2-MAGs comprise, among others, 2-palmitoylglycerol (2-PG), 2-oleoylglycerol (2-OG), 2-linoleoylglycerol (2-LG), 2-AG, 2-eicosapentaenoylglycerol (2-EPG) and 2-docosahexaenoylglycerol (2-DHG) [30].

### *N*-acylamino acids (NAAs)

NAAs belong to a large family of endogenous signalling molecules in which an amide bond covalently links an amino acid to the carboxylic moiety of a long-chain fatty acid [43]. Lately, highly sensitive mass spectrometric techniques have led to the discovery of several such lipids. Among the cluster of endogenously signalling NAAs, a few have garnered special attention from the scientific community. Namely, *N*-acyl alanines (NAAlas), *N-*acyl glycines (NAGlys), *N-*acyl leucines (NALeus), *N-*acyl phenylalanines (NAPhes), *N*-acyl serines (NASers), *N*-acyl taurines (NATaus), *N*-acyl tyrosines (NATyrs), and *N*-acyl valines (NAVals) [44]. In principle, however, each of the 20 amino acids can make an amide with the 12 major fatty acids, thus potentially leading to over two hundred NAAs.

## Characteristics and importance of eCBome mediators from a nutritional standpoint

**Fig. 3:**
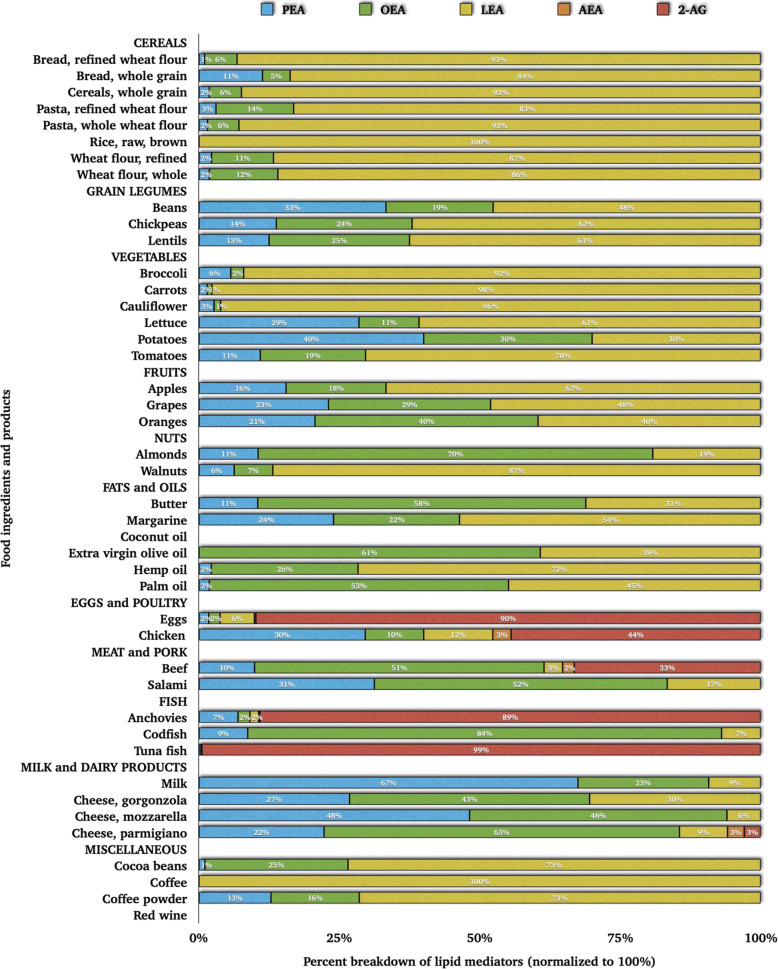
The weight percentage of *N*-acylethanolamines and 2-arachidonoyl-glycerol in various food ingredients and products [38]. **Note (alphabetical order):** 2-AG, 2-arachidonoylglycerol; AEA, arachidonoylethanolamide; LEA, linoleoylethanolamide; OEA, oleoylethanolamide; PEA, palmitoylethanolamide.

Together with 2-MAGs, bioactive lipid amides belonging to the eCBome are often detected in food items (Fig. 3) [38] and other biological samples [31]. Besides, their levels in murine tissues and human plasma are influenced by the dietary intake of the corresponding fatty acids [29, 45]. In the following section, we emphasize the most abundant and/or studied long-chain *N*-acyl amides and 2-MAGs.

### *N*-acylethanolamines (NAEs)

In the past two decades, the utilization of highly sensitive methods, such as nano-high performance liquid chromatography-tandem mass spectrometry (nano-HPLC-MS/MS), has allowed the identification and quantification of various lipid signalling mediators [46]. Henceforth, the chemical structural understanding and absolute quantification of these compounds have shed light on their relative abundance in rodent and human biological samples as well as their biological activity and molecular targets. As such, the most abundant and/or studied NAEs in human samples are the amides synthesized from saturated fatty acids (SFAs), monounsaturated fatty acids (MUFAs), and polyunsaturated fatty acids (PUFAs) of the n-6 and n-3 fatty acid families [47].

#### Palmitoylethanolamide

Biologically, PEA is always formed together with other acylethanolamides, for example OEA and LEA, and these non-endocannabinoid NAEs have a variety of biological actions, influencing feeding behavioural physiology, pain attenuation and neuroprotection [29, 48] as well as crucial effects on glucose and lipid processing [49]. PEA is derived from the most common fatty acid, i.e. C16:0, which comprises 20–30% of total fatty acids in the human body obtained either from the diet (palm tree oil, meats, milk, and dairy products) or synthesized endogenously from *de novo* lipogenesis (DNL) [50, 51]. Catabolism of PEA in the body gives rise to relatively inactive products (C16:0 and ethanolamine), causing no adverse effects [52]; therefore, its overall safety and efficacy profile makes it suitable for prophylactic use. However, the overconsumption of C16:0 due to nutritional imbalance and its adverse effects have also been reported in cardiometabolic and liver health [53], aggravating insulin resistance, metabolic dysregulation and dyslipidemia, thereby leading to abnormal fat distribution and deposition [54]. Indeed, a well-balanced dietary approach would be a key to keep a check on DNL. In addition, to derive the maximum benefits from PEA, the consumption of micronized PEA may work as an effective supplement [55]. Indeed, PEA has been suggested to play several beneficial actions in metabolic flexibility, obesity, chronic pain, and neurodegenerative, central, and peripheral inflammatory disorders through its various mechanisms of actions, including, i) synergy with eCBs in their activation of cannabinoid receptors and activation/desensitization of TRPV1 channels ("entourage effect") [56]; ii) stimulatory action on 2-AG levels via DAGL activation [57]; iii) direct activation of either PPAR-α [58] or GPR55 [24, 59]; iv) triggering the phosphorylation of adenosine monophosphate-activated protein kinase (AMPK) [49].

#### Oleoylethanolamide

OEA has gathered attention because of its peripherally acting characteristic of suppressing food intake [60]. Extensive review articles [29, 45, 61–63] have summarized the actions of OEA via oral, sub-chronic intraperitoneal- (i.p.) and intravenous (i.v.)-administration attenuating body weight gain in rodents as well as humans. In addition to its anti-obesity properties, OEA promotes lipolysis, preventing lipemia [64]. Briefly, OEA is endogenously synthesized from C18:In9 [47, 65], the production of which can further be enhanced by the intake of food products rich in MUFAs [66], especially C18:1n9 that may include n9-enriched dietary oils like olive oil, sunflower oil, peanuts and avocadoes. The peculiar trait of anorectic OEA is very distinctive of cholecystokinin (CCK), as it works in a manner that, apart from reducing the meal size, also increases the latency period [67], providing it with a unique characteristic among the class of NAEs. Even though the consumption of C18:1n9 enriched food products manifolds the synthesis of OEA and its resulting benefits, the excessive intake of oils or high fats may hinder its production because of the disruption of the gut-brain axis [68]. Therefore, a thorough probe is mandatory to ascertain the compensatory limits of the ingestion of fats and oils. The key receptors involved in the actions of OEA include, i) cluster of differentiation 36 (CD36); therefore, it is crucial to have active CD36 receptors that typically sense the long-chain fatty acids starting from the buccal cavity to produce OEA [29]; ii) activation of PPAR-α and GPR119 [69]; iii) stimulation of GPR40 [70]; iv) activation and desensitization of TRPV1 channels [71]; v) activation of the dopaminergic reward system by activating oxytocin receptors, thereby stimulating the transcription of the c-Fos gene [72]. Lately, Tovar *et al*. [73] demonstrated almost identical capacities of OEA and PEA in improving health status in diet-induced obesity. The authors showed that both compounds are equally capable of reducing weight, liver steatosis, inflammation, and dyslipidemia, giving an edge to both molecules in their inclusion and usage as nutritional supplements in treating obesity.

#### n6-acylethanolamides

LEA and AEA come under the umbrella of n6-acylethanolamides [74]. AEA, one of the two endocannabinoids together with 2-AG, belongs to the group of n6-NAEs, which also comprise the non-endocannabinoid LEA [47]. A western diet containing animal meat, eggs and dairy products is rich in n6 fatty acids, with a significant proportion of PUFA C18:2n6, i.e., an essential fatty acid that acts as a precursor for C20:4n6 and other n6 PUFAs by the action of elongation and ∆^6^-desaturase enzymes [75]. In turn, C18:2n6 and C20:4n6 serve as ultimate precursors for the on 'demand' biosynthesis and release of LEA and AEA, respectively, via the processing of the corresponding *N*-acyl-phosphatidylethanolamines [76]. LEA carries anti-obesity properties [47]; on the contrary, AEA activates CB1 receptors, thus contributing to diet-induced obesity and inflammation [76]. Today, Canada's Food Guide [77] recommends limiting SFAs ingestion. However, expansion on PUFAs consumption needs attention as higher fat consumption (45% energy) and refined carbohydrate intake may contribute to hedonic eating, thereby reducing the concentrations of anorectic LEA and increasing AEA levels [78], and posing a threat to health. Therefore, thoughtful consideration is paramount when opting for and optimizing the quality and quantity of dietary fats and oils. To derive maximum benefits from NAEs, one should not choose an imbalanced diet where PUFAs are overconsumed with respect to MUFAs. The situation may bear dire consequences for essential fatty acid deficiencies [79]. The biological activity of LEA extends by the activation of GPR119, TRPV1 and, possibly, PPAR-α and GPR55 [76]; whereas, molecular targets for AEA other than CB1 [80] and CB2 [81] cannabinoid receptors are TRPV1 channels and PPAR-γ [19, 82–84].

#### n3-acylethanolamides

Similar to n6-fatty acids, n3-fatty acids also belong to the family of essential fatty acids, which cannot be synthesised *de novo* [85]. Significant members of the n3-fatty acids are C18:3n3 and its consecutive derivatives C18:4n3, C20:5n3, C22:5n3, and C22:6n3 [86]. These fatty acids subsequently act as ultimate precursors for the endogenous synthesis of ALEA, STEA, EPEA, DPEA, and DHEA (also known as synaptamide) [87], again via the processing of the corresponding *N*-acyl-phosphatidylethanolamines. The biological activity of these amides has not been extensively studied. However, lately, their therapeutic properties have come to light [88–91]. They are proposed as neurogenetic, neuritogenetic, synaptogenic, anti-inflammatory, and anti-obesity signals, which may counteract CB1 activity and reduce lipid accretion. The most convenient way of obtaining a health advantage from these amides is by enhancing their endogenous levels through the incorporation of n3-fatty acid-enriched food items in diets that chiefly come from seafood, flaxseeds, and fats and oils derived from n3-fatty acid-enriched dietary sources. The sites of action suggested for these amides are inclusive of: i) PPAR-α [90]; ii) GPR110 [92]; iii) PPAR-γ; and iv) TRPV1 channels [87, 93].

### 2-monoacylglycerols (2-MAGs)

2-MAGs are the chief end-product of the intestinal digestion of dietary fats in humans by the action of the pancreatic lipase [94]. After ingestion of dietary fat, this enzyme hydrolyses triacylglycerols (TAGs) to produce two fatty acid molecules and one 2-MAG, wherein the portion of 2-MAG does not undergo further degradation contributing to the pool of 2-MAGs of the intestinal lumen [95]. Moreover, 2-MAGs are produced within tissues by lipolysis, as has been studied primarily concerning TAGs in adipose tissue [95]. However, 2-MAGs acting as signalling molecules are usually produced via the phospholipase Cβ-diacylglycerol lipase pathway [96]. 1,2-Diacyl-*sn*-glycerols released by the action of phospholipase Cβ on membrane phospholipids are used to generate 2-AG, which carries specific biological importance as an endogenous agonist of CB1 and CB2 receptors; 2-OG is another abundant form of the potential 2-MAGs obtained from this pathway [97].

2-MAGs are inactivated mainly by the action of MAG lipase [98], with the formation of free fatty acids and glycerol [99]. However, a ubiquitously expressed serine hydrolase ⍺/β-hydrolase domain 6 (ABHD6) is also believed to be especially important in regulating the signalling activities of 2-MAGs [100]. Inhibition of MAG lipase, by elevating the levels of 2-AG and other 2-MAGs, is of potential benefit to several disease states, such as neurodegenerative, inflammation, metabolic diseases [coronary heart/cardiovascular disease (CHD/CVD)] and cancer [101]. Additionally, inhibition of the hydrolysis of 2-AG may reduce the release of C20:4n6 to synthesise pro-inflammatory prostaglandins. The lipid signalling function of 2-MAGs other than 2-AG in the intestine takes place by activating GPR119 [102, 103], with subsequent stimulation of glucagon-like-peptide-1 (GLP-1) release and incretin action [103, 104], and of peptide tyrosine tyrosine (PYY) release and anorexigenic action [103, 104]. Overall, studies have reported that 2-MAGs other than 2-AG stimulate the incretin hormones such as gut peptides to cause a reduction in food intake and body weight gain in rats and regulate glucose-stimulated insulin secretion [105].

### *N*-acyl aminoacids (NAAs)

Acylated amino acids are also termed elmiric acids and possess, among others, analgesic and anti-inflammatory properties [106, 107]. Ingested food might directly influence the levels of thermogenic NAAs [108], although this function is yet to be elucidated thoroughly. Additionally, in both rodents and mammals, enzymes peptidase M20 domain-containing 1 (PM20D1) and FAAH interactively regulate the levels of some NAAs [109]. The most distinctive characteristic of NAAs is that they do not usually bind to cannabinoid receptors [106]. Different mammalian NAA members have various physiological implications, which are discussed below for the most studied compounds.

#### N-acyl alanines

NAAlas fetched importance because of the unique capability of some of the members of this family to increase energy expenditure in high-fat-fed mice, consequently reducing adiposity and improving glucose homeostasis [108, 110]. Therefore, these compounds may act as lipid uncouplers of mitochondrial respiration, stimulating respiration on isolated mitochondria, in cells and *in vivo* [110]. More recently, *N*-oleoyl-alanine (NOleA) reported reducing some of the signs of withdrawal from chronic or acute morphine administration in rats or from chronic heroin administration by acting via PPARα activation and indirect activation (possibly via FAAH inhibition) of CB1 receptors [111–113].

#### N-acyl glycines

NAGlys are structurally similar to AEA and are widely distributed among various mammalian tissues, including the central nervous system (CNS) [43]. The metabolism of these mediators takes place under the rate-limiting enzyme FAAH [114]. NAGlys have been suggested to bind to GPR18 [115], GPR55 [116], and GPR92 [117] for their biological actions. NAGlys can potentially be used as an important health biomarker in the medical fields of gynaecology [118] and oncology [119]. *N*-oleoyl-glycine (NOleG) has been reported to counteract some of the behavioural consequences of mild trauma in mice [120]. Importantly, this condition causes elevation of the levels of this compound in the insula [121]. NOleG also reduces nicotine self-administration as well as some of the signs of withdrawal from chronic or acute morphine administration in rats [120, 122]. PPAR-α activation and/or indirect activation (possibly via FAAH inhibition) of CB1 receptors seem to mediate these effects.

#### N-acyl serines

NASers exhibit therapeutic properties in neurology and osteology as they help in bone formation [120]. *N*-oleoyl-serine (NOleS) is the most studied member of this family of lipids [123]. Lately, a derivative of NOleS – HU-671 – was shown to reverse ovariectomy-induced osteoporosis and bone marrow adiposity more efficaciously than the endogenous compound [124]. To date, little is known about the molecular target of NASers.

#### N-acyl taurines

NATaus act as an independent category of lipids that *in vivo* are regulated by FAAH [120]. These molecules are excellent ligands for some members of the TRP family of channels, namely TRPV1 and TRPV4 [125], thereby enhancing 'glutaminergic synaptic transmission' and promoting protective effects in psychiatric disorders [126]. They have also been shown to possess wound healing properties [127].

## Influence of the diet on endogenous levels of eCBome mediators

In the previous section, we briefly discussed the biological role and mechanism of action of eCBome mediators. The following section highlights the impact of the diet on the endogenous levels of eCBome mediators.

### Dietary impact on endogenous levels of *N*-acyl amide eCBome mediators

Dietary fats and oils contain a series of bioactive lipids from the *N*-acyl amide and 2-acyl glycerol families [128] (Fig. 3). Therefore, nutritional supplementation may be a valid strategy to reduce the risk of mortality and morbidity by alleviating the severity of diseases that have been shown to benefit from treatment with these compounds.

#### Effects of diet on palmitoylethanolamide

##### Animal and in vitro studies

PEA is a naturally occurring lipid ingredient contained in foods [129]. A plethora of evidence suggests that PEA is an anti-inflammatory, analgesic, and neuroprotective mediator acting on several molecular targets in central and peripheral organs and systems [55, 102]. Bachur and colleagues [130] were the first to report the presence of PEA in mammalian tissues. PEA natural abundance and occurrence in dietary ingredients carrying beneficial medical properties make it one of the most sought-after compounds for therapeutic/medicinal purposes [131]. De Luca *et al.* [38] have successfully developed an advanced database reporting PEA concentrations and overall NAE composition in food products (Fig. 3), thus providing an easily accessible tool to opt for a natural food supplement offering benefits. In addition, whilst C16:0 intake directly influence PEA levels [33, 47, 74], concerns exist whether the beneficial effects of PEA are mediated or mitigated by its hydrolysis product, C16:0 [132]. Indeed, C16:0 carries biological effects [133]; it can also inhibit PPAR-⍺ transactivation [134], albeit with a lower potency than that needed for PEA to activate PPAR-⍺. Moreover, if C16:0 had been responsible for the effects of PEA exclusively, then a blockade of PEA hydrolysis would be expected to reduce the observed actions of PEA [132, 135]. However, an *in vitro* study conducted by Gabrielsson *et al.* [135] observed no such effect. Other *in vitro* studies [136, 137] have shown that the stereospecific esterification of C16:0 in dietary TAGs plays a crucial role in determining the endogenous levels of PEA.

##### Human studies

So far, only a few studies have addressed the association between the intake or levels of specific fatty acids with patterns of endogenous fatty acid amides in humans, particularly with C16:0 and its ethanolamide, PEA. However, numerous trials have been conducted demonstrating the safety and efficacy of PEA supplementation [55, 131]. PEA is released in the human body 'on-demand;' therefore, providing a secure niche by maintaining C16:0 tissue levels is crucial. The disequilibrium state of surplus energy intake rich in C16:0 might result in ectopic fat accumulation leading to dyslipidemia and hyperglycemia. Contrarily, ensuring optimal levels of PEA may afford therapeutic benefits against such complexities. The history of PEA takes us back to the impactful work of Kuehl and co-workers [138], which led to the first identification of this compound in egg yolks by Long and Martin [139]. The elucidation of the effect of dietary C16:0 and PEA dates back to the findings by Coburn and Moore [140], which technically was not a planned clinical trial to understand the association between the two compounds. However, the study proved to be a fundamental and foundational pillar, which led to the discovery that dietary supplementation of egg yolks could enrich tissue PEA levels. Since then, the importance of egg yolk consumption, also with regards to the supplementation of lecithin (a phospholipid), has been supported by ground-breaking work performed by Wallis [141] and Coburn *et al*. [142].

In summary, C16:0, partly through its ethanolamide, PEA, exerts multiple therapeutic functions at cellular and tissue levels, and its endogenous biosynthesis is guaranteed by DNL [50]. Therefore, the inclusion of C16:0 or PEA enriched diets contributing to increased PEA tissue levels would be the best physiological approach to gain multiple benefits by natural means. However, a necessary tab must be in place to maintain optimal dietary C16:0 intake to avoid deleterious health implications.

#### Effects of diet on oleoylethanolamide

##### Animal and in vitro studies

Evidence substantially documents that C18:1n9 acts as precursor fatty acid for the synthesis of OEA [29]. However, since oleate can be endogenously synthesized, questions arise as to whether or not there is a need to incorporate oleate-rich diets to drive OEA biosynthesis and its satiating benefits. Certainly, all mammals can convert stearate to oleate via ∆^9^-desaturases [143], a reaction that can be further enhanced by dietary copper supplementation [144, 145]. However, Bourre *et al.* [146] have demonstrated that the absence of nutritional C18:1n9 failed to maintain normal tissue levels of this fatty acid. Therefore, unquestionably, dietary incorporation of oleate is a must to achieve the optimal effective dosage of OEA to display, among others, its anorexic and hypolipidemic properties. Additionally, it has been reported that rats fed for 7 weeks not only high-fat diets but also low-fat high-sucrose (35 kcal% sucrose and 10 kcal% fat) diets exhibit disrupted feeding-dependent OEA mobilization [147]. Besides, a study conducted in mice demonstrated that a high-fat diet alters the intestinal levels of OEA [148]; however, pharmacological OEA intervention further restored physiologically lowered sensitivity to the rewarding properties of low-emulsion fat in diet-induced obese mice. For the first time, Giudetti and colleagues [149] demonstrated that OEA, along with its anti-obesity and anti-fatty liver properties, can ameliorate parameters of oxidative and endoplasmic reticulum stress in the liver of high-fat-fed rats; which *in vivo* and *in vitro* are known to be regulated, among others, by the membrane protein CD36 [29]. Notwithstanding the potent biological functions of OEA, the need to substantiate and replicate similar findings *in vivo* via feeding trials remains, and efforts should be made towards this end by investigating the compound nutritional efficacy more in-depth.

##### Human studies

Diverse pharmacological but limited nutritional data exist regarding OEA supplementation [29, 45, 61–63, 150], collating the knowledge and evidence obtained so far in the field of OEA intervention. Most of these data were accomplished using rodents, which may not translate well in humans [12]. Therefore, the nutritional/dietetics scientific community has elucidated the oral efficacy via clinical intervention feeding trials. These trials [30, 65, 151–155] set a benchmark for future clinical trials since they increased our knowledge of overall OEA effectiveness by inducing satiating properties and improving body composition post-ingestion of C18:1n9-rich dietary oils. Although all studies showed direct associations between plasma OEA and anthropometric parameters, the pharmacological trial conducted by Tutunchi *et al.* [64] indicated for the first time that OEA modulates metabolic risk factors related to non-alcoholic fatty liver disease (NAFLD). Another OEA-based pharmacological study conducted by Alireza et al. [156] showed amplified PPAR-⍺ gene expression with improved satiety scores, suggesting the need of conducting gold-standard nutritional interventional trials. The most noteworthy point of consideration while interpreting the effects of the diet on endogenous OEA levels is that most human studies measure plasma-derived OEA levels instead of intestinal OEA [157–163]. Challenging to perform invasive procedures would be required to achieve this latter measure; however, unquestionably, biopsies would more accurately reflect the picture of locally acting intestinal OEA-mediated signalling. Hence, careful considerations are warranted when interpreting the correlation/interactive analyses of OEA found in the plasma, which likely represents a composite 'spillover' from multiple organs that generate this mediator [62]. In fact, plasma OEA levels do not always correspond with tissue OEA concentrations. Nevertheless, controlled full-feeding trials [152, 154, 155] still stand out in demonstrating a shoot/boost in the circulating plasma OEA levels post C18:In9-enriched dietary oil consumption. Importantly, it has emerged that plasma OEA concentrations may be altered by a range of genetic polymorphisms [31, 70, 155], especially in genes that eventually influence the body composition, such as *CD36* rs1761667 [164], *NAPE-PLD* rs12540583, *FAAH* rs324420, *GPR40* rs1573611 and *LEPR* rs1137101.

Overall, OEA content in most mammalian organs, and less likely in the brain (which maintains stringent control of its membrane composition) [165], is responsive to the influence of changes in dietary fatty acid composition and other nutritional variables — henceforth supporting distinctive roles and functions to maintain energy equilibrium. Therefore, a balanced nutrimental plan may act as a 'vital gatekeeper' to maintain energy homeostasis by limiting energy-dense foods and increasing energy expenditure via the achievement of optimal OEA tissue concentrations.

#### Effects of diet on n6-acylethanolamides

##### Animal and in vitro studies

Mediators belonging to the eCBome are fluctuating in a time [166] and tissue-specific manner [33, 47], which can be considerably influenced by energy status, inflammation, and disease condition [105]. Diet contributes greatly to overall n6- and n3-acylethanolamides since these include in their chemical structures essential fatty acids that can solely be derived from dietary fatty acid intake [32, 33, 47, 167]. The study performed by Igarashi *et al.* [147] in rats reported that intralipid® duodenal infusion enriched chiefly with C18:2n6 increased jejunal LEA content, which went up to 10-times when rats were allowed for free-feeding. Similarly, another study conducted in rats by Alvheim and colleagues [75] showed a marked increase in liver AEA levels when C18:2n6 was fed as 8% energy for 16 weeks consecutively, leading to increased weight gain. Along the same lines, higher AEA and 2-AG concentrations were reported in salmon and mice fed a soyabean oil diet-rich in C18:2n6 [168]. Although the latter two trials focused primarily on C18:2n6 diets, they failed to report on the levels of LEA (the closest structural analogue to OEA), which is derived from C18:2n6 and stimulates GPR118 and TRPV1. Analysis of LEA could have shown the contrasting effects between LEA and AEA, showcasing the appropriate nutritional/pharmacological potential. In contrast to the previous findings, Diep and co-workers [169] reported diminished LEA concentrations post-7 days of high-fat diet feeding regardless of the diet-fed. In addition, results published by Everard and co-workers [170] noted the elevated concentrations of both LEA and AEA post-exposure to a high-fat diet for an extended duration of 5 weeks. In addition, Demizieux *et al*. [171] demonstrated the effect of n6:n3 fatty acid ratios in a more extended feeding study of 23 weeks. During the study, mice fed on the low-fat (~5 kcal% lard) diets incorporated with lard, n6-safflower oil, or n3-linseed oil for 10 weeks showed drastic modifications in tissue n6- and n3-acylethanolamides. The reported tissue levels of AEA and 2-AG evolved in an identical manner of incorporated precursor fatty acids, which was noticeable even after the mice were challenged with the high-fat (30 kcal% lard) diet in the latter half of the study (followed by another 10 weeks) except in the case of the liver. In sum, these findings suggest that fatty acids from dietary fat/oil ingestion modulate the formation of LEA and AEA, in a manner dependent on the period in life and length of exposure to the fatty acid [172]. Interestingly, the connotation of n6 and n3 fatty acids works well with their amide forms, and henceforth, the rodent studies reflect the pattern of decreased n6-acylethanolamides with the progression in n3-acylethanolamides [75, 166, 168], and vice-versa, suggesting an underlying 'push ⇌ pull' mechanism [173, 174]. Caco-2 cell lines have been extensively scanned to understand the mechanistic actions of diversified fatty acids on various pathological conditions *in vitro* [172, 175]. However, utilising an *in vitro* approach, even when used exclusively to explore the relationship between precursor n6-fatty acids and their resultant amides, lack relevance to the systemic NAE-mediated effects of the diet on the metabolic cascades of the metabolic syndrome and other disease states.

##### Human studies

Regarding fatty acids, the controversy remains between the beneficial properties of MUFAs vs PUFAs intake [29, 74], which exclusively depend on the carbon-chain length unsaturation. In energy deficit conditions, highly unsaturated fatty acids undergo β-oxidation, resulting in intestinal nitric oxide (iNO) production, thereby stimulating favourable energy expenditure. A plethora of data exists on the effects of fatty acid uptake on energy homeostasis; however, evidence is still scarce on the role of the resulting amides and their downstream actions on anthropometric-, glucose- and lipid-biomarkers. To the best of our knowledge, the dietary implications of feeding a variety of nutritional oils to humans and their direct effects have only been addressed by a limited number of studies [65, 152, 154, 155] in a full-feeding controlled fashion. The results indicated that higher intakes of C18:2n6 and C20:4n6 lead to enhanced concentrations of LEA and AEA, revealing instead decreased n3-acylethanolamides in blood plasma, with possible modulation of regional adiposity and total fat mass.

In sum, a balanced nutritional regimen with optimal n6:n3 ratios resulting in n6- and n3-acylethanolamides, through their synergistic mechanistic actions on overall health, will help attain an energy equilibrium state.

#### Effects of diet on n3-acylethanolamides

##### Animal and in vitro studies

The eCBome is an intertwined convoluted matrix whose layers are still being unfolded to decipher which signalling mechanisms regulate food intake and maintain energy homeostasis [176]. Since eCBome signalling mediators involve an array of fatty acids, it has become imperative from the nutritional and pharmacological perspective to also focus on the long- and very-long-chain n3-fatty acids (in addition to other major fatty acids) with their corresponding ethanolamides. Besides, the proven efficacy of n3 fatty acids in counteracting various diseases influencing positive health outcomes [174] spurs curiosity and demands attention to the in-depth understanding of n3-acylethanolamides. Mechoulam and his group [177] successfully isolated and synthesised DHEA and EPEA for the first time. Further adding to this knowledge, Bisogno and co-workers [178] identified DHEA as an abundant component of the bovine retina, suggesting that its occurrence was due to the high relative abundance of its precursor C22:6n3 in retinal phospholipids. Moreover, the foundational study conducted by Berger *et al.* [167] in piglets and mouse pups revealed that dietary C22:6n3 modulates brain DHEA concentrations [179]. Evolving research since then has lightened up the n3-acylethanolamids field as the past decade has resulted in very productive and fruitful data. The physiological function of the endogenous synthesis of ALEA from C18:3n3 has not been documented much, except that the latter dietary precursor directly influences rodent plasma and tissue levels [32, 33]. ALEA binds the cannabinoid receptors, albeit very weakly [180], but helps to diminish n6-acylethanolamides when feeding dietary oil combination of n3-enriched flaxseed oil and n6-rich safflower oil in proportions strongly favorable to the former. Likewise, another study conducted in mice [171] showed decreased fat mass when fed n3-enriched linseed dietary oil compared to dietary n6-rich safflower oil and lard. Although the n3-acylethanolamide levels were not analysed in the trial, a significant improvement was evident in glucose regulatory parameters in the linseed oil-fed group when put on an obesogenic diet. The findings suggest that the introduction of dietary n3 fatty acids as early as at weaning may prevent metabolic complications due to the 'compensatory effects' of n3-acylethanolamides. Whilst the biological properties of ALEA are yet to be fully explored, a significant global contribution has been obtained in understanding the beneficial properties of EPEA and DHEA [32, 33, 47, 90, 181–188]. Rodent data suggest that plasma and tissue levels of EPEA appear to be either negligible or low compared to DHEA levels and are only detectable when feeding fish oil diets enriched in C20:5n3. Besides, the endogenous synthesis of these metabolites largely depends on the form of oil provided. Rossmeisl and associates [184] demonstrated that C20:5n3 and C22:6n3, when administered in phospholipid form, result in a healthier metabolic profile than their triglyceride form, suggesting an improved bioavailability. Correspondingly, studies in obese Zucker rats [189] and high-fat diet obese mice [190] showed that krill oil (which contains C20:5n3 and C22:6n3 predominantly in the phospholipid form) could be more efficacious at reducing dyslipidemia and/or glucose intolerance than fish oil (which includes C20:5n3 and C22:6n3 mostly in the triglyceride form). However, reported effects were ascribed to decreased eCBs (AEA and/or 2-AG levels), rather than increased levels of C20:5n3- and C22:6n3-derived NAEs and 2-MAGs, which were not measured. Importantly, *in vitro* studies have shown anti-carcinogenic [191, 192], anti-inflammatory [193], anti-obesogenic [90], and neuroprotective [194, 195] properties of dietary n3 fatty acids.

##### Human studies

Clinical studies lack evidence discerning the benefits of dietary n3-fatty acids from those of their ethanolamides on metabolic parameters. Only a few clinical trials have been conducted in this area of research; however, not all such studies showed effects on human physiology [174]. A trial conducted by Berge and co-workers [196] indicated that dietary krill powder supplementation to obese participants reduced peripheral eCB hyperactivity, improving plasma triglycerides. The results suggest probable functional impact due to increased EPEA and DHEA levels; however, the study did not report such levels and was focussed on evidencing reduced AEA levels following treatment with krill oil. Similarly, an additional trial conducted by Jones and his group [197] confirmed the findings of Rossmeisl *et al.* [184], proposing that the phospholipid-based krill oil enhances the omega3-index and helps reduce n6:n3 ratios. However, again the study did not report data on n3-acylethanolamides. Besides, results from the same group recommended krill oil consumption above regular fish oil with no side effects registered [198]. These researchers assert that structural differences could play an influential role in enhancing the bioavailability of phospholipid-based krill oil over triglyceride-based regular fish oil. To understand the importance of NAEs, including the n3-acylethanolamides, the group conducted another controlled feeding trial [154], which highlighted the relationship between plasma NAE levels, body composition, and substrate oxidation in humans. The results confirmed the associations between dietary fatty acids and their circulating plasma levels with the reported increase in ALEA, EPEA and DHEA levels that blunted AEA levels. Lately, further studies [70, 152, 199] confirmed the results with reminiscent findings.

On the whole, n3-acylethanolamides show considerable versatility in counteracting multiple diseases in all their formulations. The possibility of using n3-acylethanolamides in association with other natural potent anorectic-amides could be a prospective alternative to the statins, or to other neuro-modulatory, anti-adipogenic and anti-inflammatory medicines that may carry adverse effects. Altogether, these aspects reinforce the concept that sees n3-acylethanolamides as an essential endogenous balancer for overall wellness.

### Dietary impact on endogenous levels of 2-monoacyl glycerol eCBome mediators

The mechanistic cascades regulating intestinal sensing of fats remain incompletely understood. To understand this aspect more profoundly, Kleberg *et al.* [200] conducted a rodent trial. The group developed a behavioural model of fat self-administration very much similar to the test conducted by Tellez and colleagues [148]. The findings from the trial suggest that the digestion products of fat, such as fatty acids and 2-MAGs, recruit distinct signalling pathways, likely influenced by GPR119. Additionally, in the same rats, the researchers demonstrated that fatty acids and 2-MAGs differ in reward value and their abilities to stimulate the brain reward circuitry. They, therefore, suggested that the gut-derived signals directly influence feeding, which could be modulated by a vagal-nigro-striatal pathway [148].

Furthermore, a human clinical study [201] examined the effectiveness of C8-dietary oil and olive oil in modulating gut peptide hormones. The study revealed that the 2-MAG component of dietary fat used, i.e., olive oil, strongly controlled GLP-1, PYY, and neurotensin uptake. However, both 2-OG and fatty acids (C18:1n9) upregulated fat-stimulated glucose-dependent insulinotropic polypeptide (GIP). By contrast, only the fatty acid (C18:1n9) portion of olive oil was responsible for the fat-stimulated release of CCK, which may therefore also be altered by 2-MAG hydrolysis in a tissue-specific manner following a high-fat diet [202].

To summarise, whatever fat sensors may be active during feeding, the fatty acids and 2-MAGs are likely to be sensed by different mechanisms. The long-chain fatty acid C18:1n9 is more rewarding compared to 2-OG since it may activate dopamine receptors that ultimately stimulate the histaminergic system [148, 203, 204].

## Interactions with the gut microbiome

Diet is one of the most important factors shaping gut microbiota composition [205, 206]. The gut microbiota produces, among others, a large number of microbial metabolites by metabolizing nonabsorbable dietary components [207]. Fermentation of fiber in whole grains by gut microbes can create secondary metabolites, such as short-chain fatty acids (SCFAs) – the most abundant being acetate, propionate, and butyrate – that could influence appetite and energy intake [208]. Other metabolites such as secondary bile acids produced by the gut microbiota also contribute to the regulation of energy homeostasis [209]. Notably, some gut microbiota-derived metabolites possess chemical structures similar to those of eCB-like mediators produced by the host (particularly NAEs and NAAs) and can bind to their same receptors [210, 211]. Therefore, the following section briefly describes some recently emerged results on how diet-derived 2-MAGs and NAEs influence the gut microbiota and vice versa. Other aspects of the eCBome-gut microbiome axis have been more thoroughly reviewed elsewhere [212].

The beneficial effects of PEA and OEA on the gut microbiome have been recently explored [55, 74, 212–216], and henceforth, the compound has become popular among microbiologists. Recently, Guida *et al.* [214] demonstrated that PEA (10 mg.kg^–1^, i.p.) administration to mice restored levels of *Akkermansia muciniphila* (a metabolically beneficial bacterial species, involved in energy homeostasis and inhibiting inflammation), *Eubacterium* and *Enterobacteriaceae*, thus suggesting anti-inflammatory and gut dysbiosis regulation properties. The findings are supported by Russo *et al.* [215], where PEA administration ameliorated the chronic and acute gastrointestinal tract inflammation via its action on PPAR-⍺ in the colon. Additionally, this study suggested bi-directional crosstalk between the brain and the gut microbiome, described precisely as the 'gut-microbiome-brain axis' [217–219]. OEA was suggested to bind to GPR119 receptors in the gut and hence influences the secretion of satiating hormone GLP-1, thereby alleviating cognitive deficits in patients with a mood disorder [220]. Therefore, PEA and OEA maintain the microbial symbiosis and gut barrier integrity, thereby counteracting neuroinflammatory responses, a function pertinent to adequate brain development and neurological functioning [221, 222]. Hence, PEA and OEA may act as potential supplements that may support brain and gut health simultaneously.

Comprehensive reviews [212, 223] and research articles characterize the effects of NAEs on gut microbiota, showing promising results, both in rodents [224–227] and humans [30, 228, 229]. For instance, Di Paola and colleagues [224] demonstrated gut microbiota symbiosis by sub-chronic OEA treatment in mice @ 10 mg.kg^–1^, i.p. The researchers showed that the *Firmicutes:Bacteroidetes* ratio shifted to favour *Bacteroidetes* (particularly *Bacteroides* genus) and decreasing *Firmicutes* (*Lactobacillus*), thus reducing intestinal cytokine expression. Similar observations have been confirmed by Lacroix *et al*. [230], who pointed out time-specific but weight-independent associations between AEA and DHEA concentrations and relative abundances of *Barnesiella*, *Eubacterium*, *Adlercreutzia*, *Parasutterella*, *Propionibacterium*, *Enterococcus*, and *Methylobacterium*, during progressive high fat diet-induced dysmetabolism. Another study, conducted by Manca and co-workers [226], further expands the relationship between NAEs and gut microbiota, utilizing germ-free mouse model (lacking intestinal microbiome). The study successfully showed that the gut microbiota influences intestinal eCBome signalling, significantly impacting eCBome mediators and their receptors in the intestine. Some of these mediators were also altered in the brain of germ-free mice [231]. However, in this latter case, reintroduction of the gut microbiota through faecal microbiota transfer from conventionally raised mice only partly reversed the alterations, unlike what was observed in the intestine, where the reversal was almost complete.

Another striking example of gut microbiota-eCBome crosstalk was the observation of increased intestinal DHEA and GLP-1 concentrations following *A. muciniphila* treatment of high-fat diet-fed wild type mice [226]. Previously, Everard *et al.* [227] demonstrated improved systemic inflammation (typical of high-fat diet-induced dysbiosis), with elevated intestinal 2-AG and 2-MAG congener concentrations, when mice were administered with *A. muciniphila*, thereby improving glucose intolerance and insulin resistance. Conversely, mice lacking an enzyme that degrades the 2-MAGs, and consequently exhibiting high levels of tissue 2-MAGs, were recently shown to be protected against high-fat diet-induced dysmetabolism and to exhibit at the same time potentially anti-dysmetabolic alterations in their gut microbiota, some of which could be reproduced *in vitro* following incubation of wild-type mouse faecal microbiota in culture with high concentrations of 2-AG and 2-MAGs [232].

In a context different from obesity and dysmetabolism, also NAAs have recently been suggested to produce some of their pharmacological effects by modifying the gut microbiota. A recent study [233] showed that chronic morphine or heroin withdrawal in rats could change NOleG and NOleA levels in the brain and/or the gut. These two compounds, and particularly NOleA, when administered exogenously, reduce some signs of spontaneous morphine withdrawal and naloxone-precipitated withdrawal, and this latter effect of NOleA was also accompanied by the reversal of some withdrawal-induced gut microbiota changes. Yet unpublished studies from our laboratory point to NOleG and NOleA also as possible regulators of the addictive potential of some palatable foods and obesity-induced gut dysbiosis [234].

With regards to human trials, lately an *ex vivo* study [229] demonstrated that eCBome mediators such as NAEs, and namely, PEA, OEA, LEA and AEA at 50 μM and 100 μM concentrations, profoundly influence microbial shifts with a prominent surge in *Proteobacteria* (and the family Veillonellaceae) and a decline in *Bacteriodetes*. Although the results are remarkable, the effectiveness of NAEs and their bi-directional communication with the gut microbiota in future *in vivo* studies demand attention due to the utilisation in this study of high micromolar concentrations, which may act as a limitation in human clinical trials. Nevertheless, the findings partly agree with a clinical trial conducted in humans [30], where a positive association was noted between *Veillonellaceae* in fecal samples and plasma NAE levels.

In another interesting study, oral OEA supplementation @ 125 mg twice daily in obese participants led to an increased abundance in *A. muciniphila* with significant reductions in caloric intake [228]. However, evidence [31, 235] suggests incapacitated FAAH activity in overweight and obese conditions despite resulting in potentially ineffective metabolism of both orexigenic AEA and anorexigenic OEA further investigation is warranted to inspect the potential ability of C18:1n9-OEA mediated effects via the gut-brain-liver axis. Finally, a recent study in overweight/obese individuals administered with *A. muciniphila*, either as such or in pasteurised form, showed how the beneficial metabolic effects of this commensal species were accompanied by elevation of plasma concentrations of 1- and 2-PG, a PPAR-α agonist [236].

In summary, the link between eCBome signalling and gut microbiota function, especially concerning the physiological or pathological control of energy metabolism [212], is becoming stronger and stronger with time. However, further investigation is warranted to inspect the potential ability of NAEs and 2-MAGs to regulate feeding behaviour and improve body composition parameters via their effects on gut microbiota composition.

## Future directions

The great value and knowledge accrued from the existing literature gradually have narrowed the current gaps in our thorough understanding of the eCBome. Therefore, filling these gaps, particularly to enlighten and unravel the complex eCBome, entails the involvement of biomedical researchers and clinicians in developing robust pre-clinical and clinical trials. Particular emphasis should be placed on developing acute- and long-term feeding postprandial interventional trials to enable comparisons between free- and controlled-feeding designs. Additionally, future studies must prioritize brain imaging and mapping [29] in the fasting as well as postprandial states, utilizing tracer isotopes enabling the tracking of changes in eCBome mediators within the cells as this system may change very rapidly. To this end, intestinal catheters [237] could have a supplemental advantage in reaching the intestinal segments, thus allowing the best representation of the gut-brain axis in combination with brain imaging approaches. When planning these studies, several outstanding questions need to be addressed, regarding, for instance, the anorectic and peripheral metabolism-altering properties of the lipid signalling mediators involved in eCBome. Henceforth, data emerging from such trials would spearhead the initiative to help people live healthy lives.

Additionally, pre-clinical and clinical studies should consider that both the eCBome and the gut microbiome may respond differently to distinct lifestyles and environmental conditions, thus strengthening the idea that personalised diets, instead, should become an objective to pursue. Moreover, precision nutrition would also help understand the nature of inter-individual postprandial eCBome and microbiome responses. Additionally, a plethora of evidence [238–245] suggests that physical activity/exercise acts as a strong influencer of both these 'omes,' thereby modifying their baseline signalling tone and determining whether a given diet augments too little or too much the output of one rather than the other given mediator derived from fatty acids or commensal microorganisms.

Finally, the role of other dietary macronutrients in general, and proteins in particular, possibly in synergy or in opposition with that of fatty acids, will have to be investigated in the successive trials to validate the WHEN approach. Recent data [246] have shown that whey protein supplementation reduces fasting levels of anandamide and 2-AG without promoting weight loss in pre-menopausal women with obesity on a weight-loss diet. Interestingly, whey protein has also been suggested to modify the microbiota, although not necessarily with beneficial effects [247]. Indeed, the type of source of proteins and amino acids may also be crucial because two recent studies in obese mice have shown that: i) dietary essential amino acids affect gut microbiota composition and NAA levels in the adipose tissue and plasma of obese mice, thereby enhancing energy expenditure [248]; and ii) casein proteins instead exacerbate hepatic insulin resistance due to the increased gut microbial branched-chain fatty acids [249]. Interestingly, both these effects were suggested to be mediated by the activation of mammalian Target Of Rapamycin Complex 1/S6 Kinase 1 (mTORC1/S6K1) signalling in different organs (brown adipose tissue and liver, respectively). The mTOR pathway has a vital role in coordinating energy, nutrients and growth factor availability to regulate crucial biological processes, including cellular growth, metabolism and protein synthesis through the phosphorylation of the downstream ribosomal protein, S6K1 [250].

## Summary

The bioactive lipids of the eCBome are under the strong influence of dietary fatty acids and carry potent food intake and metabolism modifying properties through multiple receptors belonging to the GPR, PPAR and TRP channel families. The activated receptors, except for CB1, trigger satiating signals or peripheral hormones, which in turn improve dysmetabolic conditions. Distinctive cascades and fatty acid precursors regulate the levels of NAEs, 2-MAGs and NAAs [251], which may signal via the gut-microbiota-brain axis. The stimulation of brain reward circuitries, such as the dopamine-histaminergic pathway [29], further enables the brain to opt for healthy fats, oil, and diets. Prolonged exposure to high-fat dietary intake may counteract endogenous anorectic pathways; for example, by acting on fat taste receptors in the gut, thus triggering orexigenic signals, promoting hedonic drive over homeostatic eating, and inducing hyperphagia. Most eCBome mediators counteract these malfunctioning pathways, whereas eCBs acting at central CB1 receptors usually contribute to them. Additionally, peripheral CB1 receptors in the liver, adipose tissue, skeletal muscle, pancreas, and kidneys instead participate in the pathological consequences of hyperphagia and obesity, thus resulting in insulin resistance [252] and dysregulated insulin release [253], hepatosteatosis [254], excessive accumulation of visceral fat [255] and renal dysfunction [256].

In this line of thought, and because the peripheral levels of different eCBome mediators with different eCBome receptors as targets depend on the dietary intake of the corresponding fatty acids, it appears that diets that are either balanced or unbalanced in their relative fatty acid content may influence the output of eCBome signalling. Thus, diets with different relative amounts of fatty acids would be resulting in either a dysmetabolism-counteracting (i.e., diets rich in MUFAs and n3 PUFAs) or -favouring (i.e., diets rich in n6 PUFAs) effects, also because of their differential impact on the bi-directional crosstalk between the eCBome and the gut microbiome, on top of their direct effects on either of these two "omes" [224, 257, 258]. Other diets rich in C16:0, through their potential to elevate the levels of two eCBome mediators carrying anti-inflammatory and anti-dysmetabolic activity, i.e., PEA and 1- and 2-PG, may also need to be re-evaluated.

## Conclusions

The diet is the central thrust area propelling eCBome actions. Ingested food unpacks multiple nutrients and will originate numerous mechanisms, thereby improving health at diversified levels. Hence, adopting the WHEN concept, with adherence to, e.g., Mediterranean and Prudent diets, would be an innovative, interactive and integrated approach for reversing metabolic syndrome and related diseases, including obesity, type 2 diabetes and NAFLD.

## Data Availability

The data in the review article are publicly available.

## Abbreviations

2-AG: 2-arachidonoylglycerol
2-DHG: 2-docosahexaenoylglycerol
2-EPG: 2-eicosapenaenoylglycerol
2-LG: 2-linoleoylglycerol
2-MAGs: 2-monoacylglycerols
2-OG: 2-oleoylglycerol
2-PG: 2-palmitoylglycerol
ABHD4: 6, 12, -⍺/β-hydrolase domain 4, 6, 12
AEA: arachidonoylethanolamide
ALEA: ⍺-linoleoylethanolamide
AMPK: adenosine monophosphate-activated protein kinase
CB1, 2: cannabinoid receptors1, 2
CCK: cholecystokinin
CD36: cluster of differentiation 36
CHD/CVD: coronary heart/vascular disease
CNS: central nervous system
COX-2: cyclooxygenase-2
DAGLs: diacylglycerol lipases-⍺/β
DHEA: docosahexaenoylethanolamide
DNL: *de novo* lipogenesis
DPEA: docosapentaenoylethanolamide
eCB: endocannabinoid
eCBome: endocannabinoidome
eCBs: endocannabinoids
EPEA: eicosapentaenoylethanolamide
FAAH: fatty acid amide hydrolase
GDE1: glycerophosphodiester phosphodiesterase 1
GIP: glucose-dependent insulinotropic polypeptide
GLP-1: glucagon-like-peptide-1
GPCRs: G-protein coupled receptors
GPR6: 18, 40, 55, 92, 110, 119, G-protein coupled receptors6, 18, 40, 55, 92,110, 119
i.p.: intraperitoneal
i.v.: intravenous
iNO: intestinal nitric oxide
LEA: linoleoylethanolamide
LEPR: leptin receptors
MAGL: monoacylglycerol lipase
MEA: myristoylethanolamide
mTORC1/S6K1: mammalian target of rapamycin complex 1/S6 kinase 1
MUFAs: monounsaturated fatty acids
NAAA: N-acylethanolamine acid amidohydrolase
NAAlas: *N*-acyl alanines
NAAs: *N*-acylamino acids
NAEs: *N*-acylethanolamines
NAFLD: non-alcoholic fatty liver disease
NAGlys: *N*-acyl glycine
NAGs: *N*-acylglycerols
NALeus: *N*-acyl leucine
nano-HPLC-MS/MS: nano-high performance liquid chromatography-tandem mass spectrometry
NAPE-PLD: N-acyl phosphatidylethanolamine phospholipase D
NAPEs: *N*-acyl phosphatidylethanolamines
NAPhes: *N*-acyl phenylalanine
NAraG: *N*-arachidonoyl-glycine
NASers: *N*-acyl serine
NATaus: *N*-acyl taurine
NATyrs: *N*-acyl tyrosine
NAVals: *N*-acyl valines
NOleA: *N*-oleoyl-alanine
NOleG: *N*-oleoyl-glycine
NOleS: *N*-oleoyl-serine
OEA: oleoylethanolamide
PEA: palmitoylethanolamide
PLD-β: phospholipase β
PM20D1: peptidase M20 domain-containing 1
PPAR-⍺/γ: peroxisome proliferator-activated receptor-⍺/γ
PUFAs: polyunsaturated fatty acids
PYY: peptide tyrosine
SCFAs: short-chain fatty acids
SFAs: saturated fatty acids
*sn*: stereospecific numbering
STEA: steariodonoylethanolamide
T2D: type 2 diabetes
TAGs: triacylglycerols
THC: ∆^9^-tetrahydrocannabinol
TRP: transient receptor channels
TRPA1: transient receptor potential of ankyrin type-1
TRPM8: transient receptor potential of melastatin type-8
TRPV1, 2, 3, 4: transient receptor potential of vanilloid-type-1, 2, 3, 4
WHEN: (Wh) olistic (E)ndocannabinoidome-Microbiome-Axis Modulation through (N)utrition
